# Relationship of Clinical Encounters to End-of-rotation Exam Scores for Fourth-year Students in Emergency Medicine

**DOI:** 10.5811/westjem.50629

**Published:** 2026-05-13

**Authors:** Max Y. Jin, Corlin M. Jewell, Daniel J. Hekman, Benjamin H. Schnapp

**Affiliations:** * University of Wisconsin School of Medicine and Public Health, Madison, Wisconsin; † University of Wisconsin School of Medicine and Public Health, BerbeeWalsh Department of Emergency Medicine, Madison, Wisconsin

## Abstract

**Background:**

Emergency medicine (EM) clerkship directors view end-of-rotation exam scores as one of the most important components in the assessment of medical student performance. Understanding factors that may impact end-of-rotation exam scores is important because strong performance during fourth-year EM clerkships is crucial for matching in EM residency. One factor that may affect exam scores is increased experience through clinical encounters. Our objective in this study was to assess the relationship between the number of clinical encounters and end-of-rotation exam scores.

**Methods:**

This was a single-site, retrospective study involving fourth-year medical students who completed a four-week EM elective between 2021–2024. We obtained exam scores and student home/away rotation status from clerkship evaluation records. The number of clinical encounters was extracted from electronic health records (EHR) via two exposure measurement methods: 1) signed notes only; and (2) signed notes or assignment to the care team on electronic EHR. We used a multivariable linear regression model to assess the impact of clinical encounters and home/away rotation status.

**Results:**

We included 108 students in this analysis. The linear regression coefficient for each clinical encounter was 0.134 (*P* = .02) and 0.089 (*P* = .09) for the two exposure measurements, respectively. Away rotation status, when controlled for the number of patients seen, demonstrated a coefficient of 2.627 (*P* = .06) and 2.464 (*P* = .08).

**Conclusion:**

The number of clinical encounters and home/away status had minimal to no impact on end-of-rotation exam scores.

## INTRODUCTION

Upon completion of each clerkship, medical students typically take the associated end-of-rotation exam to provide an objective assessment of their clinical knowledge pertinent to that specialty. Students often view this exam as the most fair form of assessment. 1 In emergency medicine (EM), clerkship directors view scores from written exams as the second most important factor when assessing student performance, only behind preceptor evaluations. 2 It is important for educators to understand what factors may influence student end-of-rotation exam scores because performance during fourth-year EM rotations is one of the most important elements for successfully matching in EM residency. 3

Many factors can contribute to student outcomes on end-of-rotation exams. 4,5 Experiential learning theory suggests that much learning occurs through reflection of past experiences and experimentation with future interactions. 6 Therefore, one factor that may be a critical determinant of medical student performance on the exam is the number of clinical encounters they have during their rotation. 7,8 There is significant variation in the number of patients seen by medical students on their EM rotation due to several factors such as location, organizational protocols, and their preceptor. 9–11 Previous literature investigating EM residency education has shown that the number of clinical encounters does not correlate with American Board of Emergency Medicine (ABEM) in-training exam (ITE) scores. 12

Additionally, no significant correlation was found when examining the relationship between clinical encounters and ABEM ITE scores for specific clinical domains. 13 However, the relationship between clinical encounters and exam scores may be different for medical students as, compared to residents, they lack substantial prior experience with common presentations to the emergency department (ED) that are the basis for the exams. Literature focusing on the impact of EM clinical exposures on exam scores for medical students has been sparse. One prior study found no difference in exam scores of students whose EM rotation occurred during COVID-19 (when fewer patients were in the ED) and students who completed EM rotations in the year prior to COVID-19. 14 The study, however, examined outcomes for their institutional exam that was similar to an Observed Structure Clinical Exam rather than a standardized written exam and was limited by small sample sizes (< 25 students in each cohort).

The correlation between number of clinical encounters and end-of-rotation exam scores has been calculated and found to be weak for other specialties including pediatrics, internal medicine, and neurology. 15–18 However, no study has examined this relationship in the unique learning environment of the EM clerkship where medical students are exposed to highly diverse conditions and are often more actively involved in patient care than in other specialties. 19 Our objective in this study was to analyze the relationship between total number of clinical encounters and end-of-rotation exam scores for fourth-year EM rotation students. Additionally, we aimed to determine whether there was any effect of home/away rotation status on exam scores, since away rotators may enter with higher baseline knowledge from previous rotations as students frequently complete their away rotation after their home rotation.

## METHODS

### Study Design and Setting

We conducted this retrospective analysis at two affiliated urban sites in the Midwestern United States. The primary site was an academic, Level I trauma ED with 54 beds that sees approximately 70,000 patients each year. The other site was a community-based ED with 20 beds that sees approximately 32,500 patients annually. The medical school curriculum is divided into three phases. Phase 1 is primarily preclinical didactics. During phase 2, all students complete a two-week EM rotation as part of their core rotations. Because Phase 2 students do not take any end-of-rotation exams for EM, they were not included in this study. Phase 3 spans the final 18 months of the curriculum, and students have the option to enroll in the EM Advanced Elective. This four-week elective is specifically designed for those interested in pursuing an EM residency and results in the generation of an EM Standardized Letter of Evaluation.

**Population Health Research Capsule**

What do we already know about this issue?

*There’s a weak, positive correlation between clinical encounters and exam scores in pediatrics, internal medicine, and neurology, but this relationship hasn’t been examined in EM*.

What was the research question?

*In fourth-year medical students, is a higher number of patients seen by medical students associated with higher EM exam scores?*

What was the major finding of the study?

*Each additional patient seen is not associated with any significant increase in exam score (B = 0.134 [p = .02] and 0.089 [p = .09])*.

How does this improve population health?

*This study suggests that clerkship directors looking to boost student exam performance should consider alternative strategies beyond pure clinical exposure*.

Other fourth-year EM electives are available for students pursuing non-EM residencies. Data from these electives were not included in this study. For the EM Advanced Elective, students complete 15 shifts in the ED where they independently care for patients under the supervision of an attending physician assigned to them at the start of each shift. The shifts are a mix of days, nights, and weekends, occurring at both sites, in a similar distribution for all students. All students have similar opportunities to see patients regardless of the site or month of rotation. Medical students were not paired with any particular attending. Each shift is 9 hours in duration, and students are expected to assist in all aspects of patient care at a similar level to early first-year residents. Their tasks include conducting a history and physical examination, calling consultants, writing notes, and dispositioning patients. Students document in the official chart and their documentation can be used for billing. 20

At the end of the rotation, students take the Society for Academic Emergency Medicine (SAEM) M4 National Emergency Medicine Exam as their end-of-rotation exam. The SAEM exam, which is free to use, was created by a national committee of education leaders in EM. 21,22 It is composed of 55 multiple-choice questions and is commonly used by clerkship directors as the end-of-rotation exam for fourth-year EM rotations. Each question was created according to the National Board of Medical Examiners item-writing guidelines. It is designed to function as an end-of-rotation, high-stakes examination and assesses a student’s knowledge on critical topics in EM. The exam is administered electronically to students and is taken asynchronously during the final week of the rotation. The structure of the EM Advanced Elective is the same throughout the course of the year, with most home students taking it in May–July and away students taking it August–October.

### Data Acquisition

We obtained end-of-rotation exam scores (reported as percentage of correct questions) and student home/away rotation status from clerkship evaluation records for the previous four years (May 2021–October 2024). The number of clinical encounters were extracted from the EHR for the same time span. We chose to use EHR data as this has been found to be more accurate than other methods at our institution. 23 Clinical encounters were included if a fourth-year medical student was identified by the EHR as involved in the patient’s care and an end-of-rotation exam score was available for that student.

We identified medical student engagement with the clinical encounter via two methods. The primary exposure measurement identified included encounters where notes were signed by a medical student, as this reflects definite student involvement in the patient’s care. The secondary exposure measurement included cases where medical students either signed a note or were assigned to the patient’s care team in the EHR. The inclusion of care team additions added sensitivity but at the expense of specificity, as sometimes students will sign up for the wrong patient or the situation in the ED changes such that the student does not actually manage that patient. We analyzed the relationship between patient encounters and end-of-rotation exam scores for both exposure measurements. Exclusion criteria included students completing their two-week EM rotation during their core clinical rotations or other EM rotations outside the EM Advanced Elective. All student names were de-identified, and no individual patient-level data was extracted to maintain anonymity.

### Data Analysis

We used two multivariable linear regression models to assess the relationship between the number of clinical encounters and end-of-rotation exam scores. One model used the primary exposure measurement values for clinical encounters, while the other model used the secondary exposure measurement values. Predictor variables included in our models were number of clinical encounters and home/away rotation status. For all statistical analyses performed, a *P* value ≤ .05 was taken as statistical significance. We analyzed data using IBM SPSS Statistics v30 software (International Business Machines Corporation, Armonk, NY).

This study was evaluated by the University of Wisconsin Institutional Review Board and deemed to be exempt from full review as quality improvement.

## RESULTS

We identified 110 medical students as having an end-of-rotation exam score available. Two of these students did not have any clinical encounters found in the EHR and were excluded. For the 108 medical students included in this study, the average (standard deviation [SD]) number of clinical encounters during this four-week elective was 54.70 (11.39) based on signed notes only (Table 1). When also including encounters where students assigned themselves to the care team, the average (SD) number of encounters was 60.30 (13.02). The average end-of-rotation exam score was 79.12% (6.98). Our linear regression model found that for each additional patient seen, exam scores increased by 0.134% (95% CI, 0.018–0.249; *P* = .02) and 0.089% (95% CI, −0.013 to 0.191; *P* = .09) based on the two exposure measurements, respectively. The *R*^2^ for the model using the primary exposure measurement was .070, and the *R*^2^ for the model using the secondary exposure measurement was .050.

**Figure 1:**
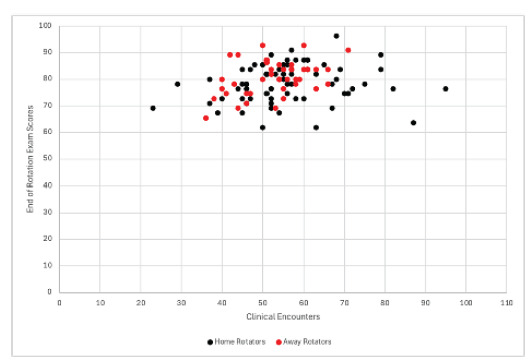
Scatterplot of clinical encounters and end-of-rotation exam scores (primary exposure measurement).

**Figure 2:**
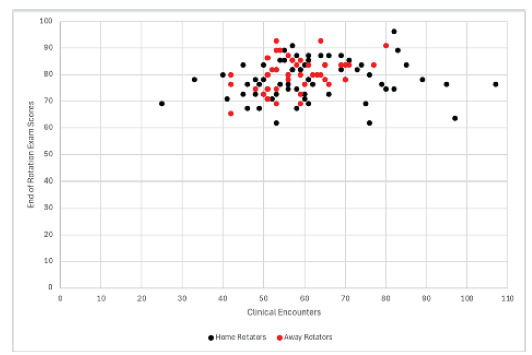
Scatterplot of clinical encounters and end-of-rotation exam scores (secondary exposure measurement).

Of the 108 medical students included in this study, 67 were home rotators who had an average (SD) end-of-rotation exam score of 78.29% (7.24) (Table 1). Home students saw an average of 55.99 (12.84) to 61.52 (14.97) patients based on the two exposure measurements. The other 41 students were away rotators with an average exam score of 80.47% (6.41). These students saw an average of 52.61 (8.24) to 58.29 (8.77) patients. Away students scored higher on end-of-rotation exams by 2.627% (95% CI, −0.074 to 5.329; *P* = .06) and 2.464% (95% CI, = −0.258 to 5.185; *P* = .08) when adjusted for the number of patients seen. A scatterplot displaying each individual student’s number of clinical encounters and end-of-rotation exam scores is presented in Figure 1 and Figure 2 for the two exposure measurements, respectively.

## DISCUSSION

Our results indicate that the number of clinical encounters had minimal to no impact on end-of-rotation exam scores in EM. Being an away rotator may be associated with higher exam scores, although these results were not statistically significant. Based on our data, students saw 3–4 patients per 9-hour shift. While this appears low, it is not an unreasonable number for trainees who are new to the clinical environment. It has been previously found at our institution that more junior-level medical students have low overall exposure to the highest acuity patients. 11 This may contribute to fewer patients seen due to the high acuity and complexity within our patient population. Furthermore, students are still adapting to the new intern-level responsibilities that are tasked to them during this rotation.

Our linear regression results (B = 0.134 and 0.089) suggest a trend of a weak, positive relationship between clinical encounters and end-of-rotation exam scores, although this did not reach statistical significance. The positive direction of this relationship has also been reported through previous studies analyzing correlations in pediatrics (*r* = 0.189), 17 internal medicine (*r* = 0.01 – 0.17 for four groups based on what quarter they completed the rotation), 18 combined internal medicine/pediatrics (*P* = .17), 15 and neurology (*r* = 0.142). 16 It is plausible that the utility of clinical engagements may be greater for students who have a higher level of baseline clinical knowledge, which could explain why away rotators in our study (who are on their second or third rotation) scored higher on the end-of-rotation exam. Understanding factors that influence EM rotation achievement is important because it can aid in future curriculum development; more training time should be considered for components that most positively influence student success.

Previous studies have indirectly assessed the relationship between clinical encounters and exam outcomes. Overall, studies examining the relationship between clinical encounters and end-of-rotation exam scores have been mixed, highlighting the need for further investigation into how multiple factors in the clinical environment can interact to influence student learning. In EM, it has been reported that crowding in the ED was negatively associated with exam outcomes for a two-year sample. 4 Of the studies examining other specialties, only one study of a psychiatry rotation reported a significant relationship with exam scores when comparing different lengths of rotation. 24 In surgery, it was found that students who completed the clerkship later in the curriculum (ie, completed clerkships for other specialties first and had more overall clinical experience) achieved better exam scores, although no statistical analysis was performed. 25 No significant differences were found in end-of-rotation exam scores for pediatrics when compared to a course without clinical activities and obstetrics/gynecology across different clinical hours worked, unless the longer hours occurred in the final two weeks of the clerkship. 26,27

It is important to recognize that engagement with clinical encounters is just one aspect of a student’s learning experience. Other potential determinants of end-of-rotation exam success have been previously investigated. In psychiatry, attendance at a comprehensive review session three days prior to the exam was found to result in significantly increased exam scores. 28 A study in surgery found that use of outside resources was correlated with better exam scores. 29 Specifically, it was found that the use of four different resources was optimal, with question banks and focused review textbooks being the most effective options. Regarding time spent studying, it has been found that 6–10 hours and 11–15 hours are ideal for the first and second half of the surgical rotation, respectively. The use of practice exams has also been associated with higher exam scores across several specialities. 30 This suggests that clinical knowledge and test knowledge may be different constructs as common topics for exam questions may be rarely seen in the clinical environment (eg, toxins/antidotes).

Future studies would benefit from a prospective design, so that the number of clinical encounters can be more accurately identified and potential confounding variables such as study time and study materials can be accounted for. It would also be of interest to examine the relationship for other standardized end-of-rotation exams, such as the NBME EM Advanced Clinical Exam. Lastly, the effect of home or away rotation status on clinical exposures warrants further investigation. 31

## LIMITATIONS

Our study has several important limitations. During the extraction of clinical encounters, it is possible that there were cases where a medical student was involved in a patient’s care but did not write a note or assign themselves to the patient’s care team. The specifics of on-shift teaching and observation of other cases is hard to account for in a retrospective study. A student may have been asked to help with the initial evaluation of a critically injured trauma patient or observe a unique case but were not the part of the primary team involved in that patient’s care. While these cases would not be reflected in our analysis, as the chart would not indicate the medical student’s involvement, they were sstill likely to be important learning experiences that could help them answer end-of-rotation exam questions.

Another limitation was that we were unable to collect data on potential other confounding variables such as U.S. Medical Licensing Exam test scores or prior grades, which would have been hard to control for as much of the preclinical work is graded pass/fail. While we recognize that test-taking ability contributes to exam outcomes, students still need EM knowledge to be successful on the end-of-rotation exam. Neither did we measure the breadth of chief complaints seen, other study resources used, or the amount of time spent reviewing clerkship content, which could have been additional mediating variables. Lastly, because we focused on the SAEM M4 exam our study results may not be directly applicable for programs that use other exams.

## CONCLUSION

The relationship between the number of clinical encounters recorded by medical students on their emergency medicine rotation and their end-of-rotation exam scores was minimal to none. Similarly, the effect of home vs away rotation on exam scores was also negligible.

## Figures and Tables

**Table 1:** Number of medical student clinical encounters and their end-of-rotation exam scores in a study assessing the relationship between clinical encounters and exam outcomes.

Rotation status	Number of students	Mean number of clinical encounters (SD) - primary exposure measurement	Mean number of clinical encounters (SD) - secondary exposure measurement	Mean end-of-rotation exam score (SD)
Home and Away	108	54.70 (11.39)	60.30 (13.02)	79.12% (6.98)
Home	67	55.99 (12.84)	61.52 (14.97)	78.29% (7.24)
Away	41	52.61 (8.24)	58.29 (8.77)	80.47% (6.41)

*SD*, standard deviation.
